# Visceral leishmaniasis cyclical trends in Bihar, India – implications for the elimination programme.

**DOI:** 10.12688/gatesopenres.12793.1

**Published:** 2018-02-21

**Authors:** Rinki M Deb, Michelle C Stanton, Geraldine M Foster, Rudra K Das Gupta, Nupur Roy, Pradeep Das, Akshay C Dhariwal, Michael Coleman

**Affiliations:** 1 Vector Biology Department, Liverpool School of Tropical Medicine, Liverpool, L3 5QA, UK; 2 Faculty of Health and Medicine, Lancaster University, Lancaster, Lancashire , LA1 4YW, UK; 3 National Vector Borne Disease Control Programme, Directorate General of Health Services, Ministry of Health and Family Welfare, Delhi, 110054, India; 4 Rajendra Memorial Research Institute of Medical Sciences, Patna, Bihar, India

**Keywords:** Visceral leishmaniasis, kala-azar, climate, India, Bihar, elimination

## Abstract

**Background:** Visceral leishmaniasis (VL) is a vector-borne disease of public health importance in India, with the highest burden of disease in the states of Bihar, Jharkhand, West Bengal and Uttar Pradesh. The disease is currently targeted for elimination (annual incidence to less than one per 10,000 population) using indoor residual spraying, active case detection and treatment. Historically the disease trend in India has been regarded as cyclical with case resurgence characteristically occurring every 15 years.  Understanding this pattern is essential if the VL elimination gains are to be sustained. To better understand the cyclical trends, annual climatic indicators including rainfall, temperature and humidity over time were compared with annual VL case incidence data.

**Methods: **Annual climate data (rainfall, average and maximum temperature and specific humidity) from 1956-2004 were used to identify potential factors influencing VL incidence.  Months relevant to the VL life-cycle were identified and defined (Monsoon, Sand-fly Peak, Pre-Sand-fly Peak and Annual) for analysis. The Kruskall-Wallis test was used to determine significant difference between categorical rainfall and VL incidence, whilst univariate negative binomial regression models were used to determine predictors of disease incidence.

**Results:** The negative binomial regression model showed statistically significant associations (p <0.05) for VL incidence and maximum temperature, and average temperature, when considering annual and pre-sand fly peak time periods. No other associations between humidity, rainfall or temperature and VL incidence were detected (all values p >0.05).

**Conclusion:** The VL programme in Bihar has made significant progress in adopting best practices for improved treatment and vector control, with the aim to achieve VL elimination.  However, open access granular programme data for indoor residual spray activities and case detection is required to fully understand the role of climate in disease transmission and potential resurgence.

## Background

Visceral leishmaniasis (VL) is a tropical disease of public health importance in India. Caused by *Leishmania donovani* parasites and transmitted by the sand fly *Phlebotomus argentipes*, the disease is anthroponotic in India and endemic in four States: Bihar, Jharkhand, West Bengal and Uttar Pradesh ( Addy & Nandy, 1992). It is estimated that 90% of VL cases in India originate from Bihar ( Singh *et al.*, 2010). In 2005, India with Bangladesh and Nepal, set a target to eliminate VL with incidence at less than one case per 10,000 population by 2015, which was subsequently readjusted to 2017. This is in line with the London declaration on Neglected Tropical Diseases (NTDs), which set the aim to eliminate VL as a public health problem by 2020.

The seasonal and interannual scale climate trends seen in vector-transmitted diseases have been widely reported ( Connor *et al.*, 1999). In India, malaria prediction was first conducted using climatic and socioeconomic data in the 1910s by Captain S. R. Christophers from the British Army ( Gill, 1938). This system was implemented until the 1940s, when malaria was not seen as disease of public health concern within the Indian subcontinent ( Gill, 1938). Despite the burden of VL disease in India, little work has been done to associate climatic indicators with case incidence. Historical trends have previously shown that there is a resurgence of VL every 15 years post control: whilst widely accepted, there is limited understanding about the cause of this pattern ( Malaviya *et al.*, 2011; Muniaraj, 2014).

Napier first proposed that VL in India was cyclical in nature in 1946, before the suspected epidemics of 1977 and 1991. Visceral leishmaniasis epidemics in Assam (1875 and 1950) however were thought to be caused by the influenza pandemic of 1918–1919, malaria, famine and earthquakes ( McCombie Young, 1924; Napier, 1943; Rogers, 1908). Findings from McCombie Young (1924) in Assam, supported this and after the introduction of antimonial drugs, a dramatic decrease in case mortality from 90% to 10% was observed ( Dye, 1992; McCombie Young, 1924; Muniaraj, 2014). More recently, Muniaraj attempted to elucidate the cause behind the 10–15 year cycle, attributing the VL case trend to vector control practices and the need for appropriate therapeutic strategies to reduce VL mortality ( Muniaraj, 2014). However, Dye has referred to this cyclical trend of *L. donovani* VL as “part of the folk wisdom of tropical medicine”, suggesting that post-kala-azar dermal leishmaniasis (PKDL) patients serve as a parasite reservoir: chronically infective and available to sand flies ( Dye, 1992; Rogers, 1908). Studies by Addy and Nandy have suggested that disease transmission during the suspected epidemic period of 1977 in Bihar, was most likely passed on person to person from active VL cases ( Addy & Nandy, 1992).

Associations between climatic indicators, vector density and VL incidence in India have previously been detected when analysing district level data ( Bern *et al.*, 2010; Dhimal *et al.*, 2015; Malaviya *et al.*, 2011; Tiwary *et al.*, 2013). Generalised associations for Bihar as a State, currently do not exist. Humidity, temperature, rainfall, soil temperature and moisture are all widely accepted factors that influence the development of *Ph. argentipes* ( Bhunia *et al.*, 2010). There are two seasonal peaks of *Ph. argentipes* in Bihar, one between March to June and another in October to November ( Dinesh *et al.*, 2001; Picado *et al.*, 2010). Associations have also been made between VL incidence and air temperature, relative humidity and annual rainfall in the Gangetic plain, which includes regions within Bihar ( Bhunia *et al.*, 2010). Malaviya *et al*. noted seasonality of VL case trends, with peaks in March to April and a minor secondary peak in July ( Malaviya *et al.*, 2011). In addition, the onset of disease symptoms were recorded, in descending order, from April to June, June to September and October to December ( Perry *et al.*, 2013). This paper investigates the impact of annual climatic and disease trends on the cyclic nature of VL burden in Bihar.

## Methods

### Study area

The State of Bihar is located in the eastern part of India, extending over 94,163 km and averages 52.73m above sea level. It is entirely land-locked, bounded by Nepal in the north and the State of Jharkhand in the south. To the east lies the humid State of West Bengal and to the west, the sub-humid State of Uttar Pradesh. There are 38 districts in Bihar and the total population, according to the last census in 2011 census, was 104,099,452.

## Data sources

For full details on data sources accessed for this research study, see supplementary Table 1.

### Epidemiological data

Yearly case numbers from Bihar were compiled using open access data available from the National Vector Borne Disease Control Programme (NVBDCP), Government of India ( NVBDCP n.d; NVBDCP n.d.b; Kala-azar or Visceral Leishmaniasis) and published literature quoting historical case data from Government of Bihar (GOB) and Government of India records. Case numbers obtained from the NVBDCP originate from primary health care facilities and hospital records within Bihar, where diagnostic testing, followed national guidelines ( NVBDCP n.d.). Annual incidence was calculated using population totals obtained from Bihar population data, obtained from decennial census surveys conducted by Ministry of Home Affairs, Government of India ( Directorate of Economics & Statistics, 2011). Annual population growth statistics from the Directorate of Economics and Statistics, Patna, Bihar (1956–1959) and the World Bank Databank (1961–2014) was used to produce yearly population figures between census surveys ( by multiplication of annual growth rate and population for previous year). Census numbers for Bihar were taken directly where available. Visceral leishmaniasis incidence was calculated per 10,000 for all years where case and total population data were available.

### Indoor residual spraying (IRS) data

Peer-reviewed literature searches were used to determine years when IRS was performed in Bihar between 1931–2014 (Date of last search: 31 November 2015). The search term strings included “visceral leishmaniasis”, “kala-azar”, “indoor residual spraying”, “IRS”, “India” and “Bihar” were used on PubMed and Google search engines to establish historical spray activities. Bibliographies from relevant peer-reviewed journals were used to identify additional information and data.

### Rainfall data

Bihar State historical monthly rainfall data (1871–2013) was obtained at the time of analysis from the Indian Institute of Tropical Meteorology. Adapted Earth System Science Organisation (ESSO)-India Meteorological Department (MoES) long period average (LPA) rain fall categories were used to classify rainfall data for the selected time period into standardised groups: *Deficient* = rainfall below 80% LPA, *Below Normal* = rainfall 80–90% LPA, *Normal* = rainfall 90–110% LPA, *Above Normal* = rainfall 110–120% LPA and *Excess* = rainfall above 120%.

### Temperature and humidity

The average and maximum temperature and specific humidity data corresponding to the coordinates of Bihar were extracted from satellite sources and digital databases through the International Research Institute for Climate and Society website. Data for Bihar, accessed through the website, originated from the National Centers for Environmental Prediction (NCEP) and the National Centre for Atmospheric Research (NCAR), as part of the NCEP/NCAR Climate Data Assimilation System (CDAS) Reanalysis Project. Daily data provided at a 1 degree spatial resolution were aggregated to provide a monthly output for the entire State. Temperature data was converted from Kelvin to degrees Celsius.

### Climate periodicity

In order to aggregate monthly climate data into an appropriate annual indicator, literature searches were conducted and two clear time periods were identified as the most relevant for VL transmission: *Monsoon Season* (June-September), and *Sand Fly Peak* abundance (spanning months March - June and October - November) ( Dinesh *et al.*, 2001).

As the sand fly life cycle is estimated to take approximately one month, a one month window prior to sand fly abundance peaks (spanning months February-May and September-October), categorised as *Pre-Sand Fly Peak*, was also considered relevant in determining risk factors associated with VL transmission.

Finally, the *Annual* category for all variables was used to identify any trends between annual VL incidences and calendar year annual climatic variables.

### Analysis

Monthly data from the specific climatic periods ( *Annual, Monsoon Season*, *Sand Fly Peak* and *Pre-Sand Fly Peak*) were averaged to produce a single annual figure per time period. A non-parametric Kruskal-Wallis Test was performed to determine whether VL incidence varied between the different annual rain categories (deficient, below normal, normal, above normal, excess). Univariate negative binomial regression models were fitted to the VL incidence data for each of the climatic indicators (specific humidity, average and maximum temperature) using data from the periods 1956–1960 and 1977–2013. To account for multiple testing, the resulting p-values were adjusted using the false discovery rate (FDR) correction ( Benjamini & Hochberg, 1995). Climate variables with a p-value less than 5% were significant, plus the Akaike Information Criterion (AIC) values were compared to determine which of the models was the best fit to the data. To account for long-term changes in climate and VL incidence, the analysis was repeated using temporally continuous data only (1977–2013) using climate anomalies (observed values – period average) and a linear temporal trend as predictors in the negative binomial regression models. Analysis was conducted in R 3.2.5, SPSS (Version 22) and Graphpad Prism (Version 6.07).

## Results

### Epidemiological data

The earliest record of case data available for Bihar was in 1934, however review of the historical literature also showed a disparity in the total number of cases reported between 1934 and 2014. A total of 1,190,166 cases were recorded when using GOB only records( NVBDCP n.d; NVBDCP n.d.b; Sanyal *et al.*, 1979). Conversely, 1,461,963 cases were recorded when including GOB records and independent surveys (GOB&I) data conducted through National Institute of Communicable Diseases, Delhi (1977), and Government of India under the Malaria Department (1991).

**Figure 1.**
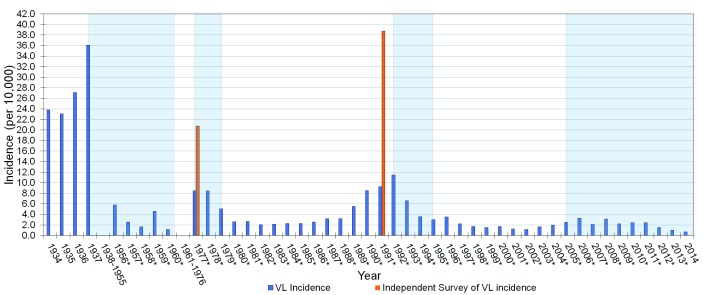
Historical Overview: Annual VL Incidence in Bihar and IRS activities. The dark blue bars represent the annual VL incidence per 10,000, as reported by the VL Programme (GOB only). In the absence of historical population at risk data, the total population in Bihar was used to calculate incidence. Independent surveys conducted in 1977 by National Institute of Communicable Diseases, Delhi and GOB and 1991 by Government of India under the Malaria Department, reported VL incidence per 10,000 is shown in orange for those two years (GOB&I). Light blue shading represents years where IRS was ongoing, whilst white represents years where IRS was stopped. Years marked with an asterisk were used in analysis of climatic, case and IRS indicators.

Records of VL cases were unavailable between 1938–1955 and 1961–1976, most likely coinciding with public health priorities moving away from VL in Bihar. However, case records were available consistently from 1977 to present ( Figure 1). As noted by Bora, “due to limited surveillance strategies” information on outbreaks before 1977–78 was patchy ( Bora, 1999). Cases recorded were largely confirmed by tissue specimen microscopy, however since 2005 the NVBDCP guidelines advise use of the rk39 rapid diagnostic test. Other methods such as direct agglutination test, enzyme-linked immunosorbent assay, parasite culture and PCR may have been used at a smaller scale to diagnose cases presented in health facilities associated with research institutes.

GOB VL incidence data, collected solely through Primary Health Centres, showed the highest VL incidence was in 1992 (11.480 per 10,000 (75,523)) ( Figure 1 & Table 3). The second highest VL incidence was recorded in 1991 (59614 cases (9.238 per 10,000). Bora reported that a further peak in VL cases occurred in 1974, however, as the programme assumed elimination had been achieved and stopped VL case data collection, no information was available to calculate incidence ( Bora, 1999). The lowest incidences recorded were seen in 2014 (7615 cases - 0.705 per 10,000) and 2013 (1.005 per 10,000 (10730 cases) ( Figure 1 & Table 3).

According to GOB&I data, the highest annual case reports were recorded in 1977 (100,000 cases – incidence of 20.720 per 10,000) and 1991 (250,000 cases – incidence of 38.741 per 10,000) ( Figure 1). GOB data only was used in the analysis of climatic indicators.

### IRS data

IRS was first adopted for VL elimination in Bihar through the National Malaria Eradication Programme (NMEP) in the 1940s ( Thakur, 1984a). Activities were stopped 1962–76 in response to a rapid fall in VL case numbers, however resumed for two years again in 1977, with support from Government of India, to combat the outbreak declared that year ( Muniaraj, 2014; Thakur, 1984a). Following a survey in 1991, an outbreak of VL was declared and IRS was conducted 1992–1995, after which IRS was stopped ( Thakur, 2007). In 2005, with the launch of the “Kala-azar Elimination Programme”, IRS was restarted to reach the elimination target (less than one in 10,000 within a primary healthcare centre/block) by a recently revised deadline of 2020 ( Mondal *et al.*, 2009; WHO, 2016). No records of IRS activities were available during years 1934–1936, when there was high case burden, and for the VL peak of 1974 ( Bora, 1999).

### Rainfall

Sixty-two years of rainfall data, 1951–2013, were available to determine trends and association between rain time categories and VL incidence, however due to gaps in VL incidence data (1951–1955 and 1961–1976), a total of 42 matched years (1956–1960 and 1977–2013) were used to complete the analysis (marked with an * in Figure 1). The average ( *Annual)* rainfall for the total 42 matched years was 1017.66mm. The average rainfall during *Monsoon Season* periods for the same matched years was 2517.92mm. When considering the sand fly-related time categories for these years, the average rainfall during *Sand Fly Peak* months was 586.46mm and 659.01mm during the *Pre-Sand Fly Peak* months.

After categorising the data into the five standardised groups, the rainfall during *Annual* and *Monsoon Season* time periods were found to be mostly *Normal* to the LPA (18/42 (42.86%) and 14/42 (33.33%) respectively). During the *Sand Fly Peak* period, rainfall was classified most frequently as *Deficient* to the LPA (13/42 (30.91%)). Conversely, when considering the *Pre*- *Sand Fly Peak* period, rainfall was found to be *Excess* to the LPA most frequently (12/42 (28.57%)).

### Temperature and humidity

A total of 66 years of temperature and humidity data was available from data sources, however, given the limited availability of VL incidence data, a total of 43 years of temperature and specific humidity data (1956–1960 and 1977–2014) were used. As shown in Table 2, the mean average temperature over the different time groupings ranged between 21.74 and 25.36°C whilst the mean maximum temperature was 28.36–31.14°C. Furthermore, the mean specific humidity (x1000) ranged between 12.000 – 20.000kg/kg.

### Climatic variables and VL incidence

Rainfall during 1992, the highest VL incidence according to GOB data, was classified as *Deficient* during all time periods ( Table 3). During the year of the second highest VL incidence, rainfall was classified as *Below Normal* to the LPA when considering the *Annual* and *Monsoon Season* time periods ( Table 3). During *Sand Fly Peak* and *Pre-Sand Fly Peak* time periods, rainfall was *Deficient* to the LPA.

The lowest VL incidence was recorded in 2014, however no rainfall data was available for this time frame. VL incidence was recorded as low in 2013 (1.005 per 10,000) during which, rainfall was classified as *Deficient* during the *Monsoon Season*, *Excess* during the *Pre-Sand Fly Peak* and *Sand Fly Peak*, and *Normal* for the *Annual* ( Table 4).

When considering VL incidence and rainfall together, no statistically significant relationship was detected for any of the time periods ( *Annual* (p=0.265), *Monsoon Season* (p=0.281), *Sand Fly Peaks* (p=0.602) and *Pre-Sand Fly Peak* (p=0.416)) ( Table 4). Overall, average VL case incidence, irrespective of time period, was lower (2.172–3.133) during years of higher rainfall (Above Normal or Excess to the LPA).

The average VL incidence when considering all 22 IRS years, between 1956 and 2014 ( Table 1), was 3.590. For the 21 years when IRS stopped, the average VL incidence was 3.000. The negative binomial regression model fitting identified statistically significant negative associations between both *Average Temperature* and *Maximum Temperature* and VL incidence (GOB data) during the *Annual* ( *RR= 0.682, p=0.0186 and RR=0.667, p=0.003 respectively)* and *Pre-Sand fly Peak (RR=0.796, p=0.0494 and RR= 0.814, p=0.0501 (close to statistically significant) respectively)* time periods only ( Table 2). However, after correcting for multiple testing using the FDR correction, only Annual Maximum temperature was significant (p=0.0360). Of these regression models, Annual Maximum temperature was the best fitting model as assessed using AIC. No other significant associations were detected for temperature and other time periods, or humidity and VL incidence ( Table 2). Summaries of the models fitted to climate anomalies for the period 1977–2013 can be found in Supplementary Table 2. After adjusting for a decreasing temporal trend in incidence, only annual specific humidity anomalies were associated with incidence (p=0.0398) however after adjusting for multiple testing this association became non-significant (p=0.4776).

**Table 1.** Overview of IRS activities for VL in Bihar, India.

IRS	Years	Programme Name	Programme led by
1	1937–1962	National Malaria Eradication Programme	National-run Programme
2	1977–1979	Kala-azar Control Programme	National Institute of Communicable Diseases with UNDP assistance
3	1992–1995	Kala-azar Control Programme	
4	2005–Present	Kala-azar Elimination Programme	State-run Programme

**Table 2.** Temperature and Humidity descriptive for all time periods. Negative Binomial model with VL incidence using data from GoB only.

		Annual	Monsoon Season	Sand Fly Peak	Pre-Sandfly Peak
Average Temperature	Mean (°C )	21.74	25.36	23.73	22.99
	Range (°C )	20.88-23.25	24.05-26.77	22.85-25.78	21.95-24.60
	s.d.	0.551	0.446	0.684	0.782
	Coefficient	0.6818	0.8708	0.8383	0.7959
	95% CI	0.5040 - 0.9355	0.5127 - 1.4919	0.6541 - 1.0914	0.6454 - 0.9890
	p-value	0.0186 *	0.5142	0.1788	0.0494 *
	Adjusted p-value	0.1116	0.6856	0.3576	0.1503
	AIC	927	932	931	929
Maximum Temperature	Mean (°C )	28.36	28.75	31.14	30.18
	Range (°C )	27.30-29.75	27.21-30.81	29.89-32.84	28.78-31.97
	s.d.	0.645	0.645	0.796	0.866
	Coefficient	0.667	0.9466	0.865	0.8137
	95% CI	0.5055 - 0.8863	0.6656 - 1.3574	0.6793 - 1.1086	0.6660 - 0.9977
	p-value	0.003 *	0.709	0.2174	0.0501 ^¤^
	Adjusted p-value	0.0360 *	0.8508	0.3727	0.1503
	AIC	926	933	931	929
Specific Humidity (x1000)	Mean (kg/kg )	13	13	13	13
	Range (kg/kg)	12.000 - 13.000	18.800 - 20.000	11.000 - 13.000	10.000 - 13.000
	s.d.	0.00033	0.00031	0.00056	0.00053
	Coefficient	0.6664	0.9664	0.9845	0.8296
	95% CI	0.4121 - 1.0313	0.6600 - 1.4013	0.7431 - 1.3011	0.5784 - 1.1716
	p-value	0.0813	0.8586	0.9165	0.2811
	Adjusted p-value	0.1951	0.9165	0.1965	0.4217
	AIC	930	933	933	932

* P-value is significant at the 0.05 level. P-value is nearly significant. (Akaike Information Criterion (AIC), Confidence interval (CI), standard deviation (s.d.))

**Table 3.** Rainfall, VL case incidence per 10,000 (GOB data) and Kruskal-Wallis analysis. (Average temperature (Ave. Temp), Maximum temperature (Max. Temp), Specific Humidity (Specific Hum.))

Year	VL incidence	Annual				Monsoon Season				Sand Fly Peaks				Pre-Sand fly Peaks			
		Ave. Temp (°C)	Max. Temp (°C)	Specific Hum. (kg/kg)	Rainfall	Ave. Temp (°C)	Max. Temp (°C)	Specific Hum. (kg/kg)	Rainfall	Ave. Temp (°C)	Max. Temp (°C)	Specific Hum. (kg/kg)	Rainfall	Ave. Temp (°C)	Max. Temp (°C)	Specific Hum. (kg/kg)	Rainfall
1991	9.238	21.64	28.34	0.013	Below Normal	25.64	29.09	0.020	Below Normal	23.62	31.47	0.012	Deficient	23.05	30.59	0.012	Deficient
1992	11.480	21.12	27.97	0.012	Deficient	25.39	29.23	0.019	Deficient	23.44	31.29	0.011	Deficient	22.09	29.75	0.011	Deficient
2013	1.005	22.38	29.38	0.013	Normal	22.38	28.93	0.020	Deficient	24.63	32.31	0.012	Excess	24.42	31.92	0.012	Excess
2014	0.705	22.46	29.75	0.013	N/A	22.46	30.81	0.020	N/A	24.56	32.83	0.011	N/A	23.55	31.17	0.012	N/A

**Table 4.** Annual and Maximum Temperature, Humidity and Rainfall during high and low VL incidence. VL incidence based on data from GOB data. (Minimum (Min.), Maximum (Max.), Average (Ave.)

		Annual						Monsoon						Sand fly Peaks						Pre Sand fly Peaks					
Rainfall Category	Description	Rainfall Range/ mm	# Years	Incidence per 10,000			P value	Rainfall Range/ mm	# Years	Incidence per 10,000			P value	Rainfall Range/ mm	# Years	Incidence per 10,000			P value	Rainfall Range/ mm	# Years	Incidence per 10,000			P value
				Min.	Max.	Ave.				Min.	Max.	Ave.				Min.	Max.	Ave.				Min.	Max.	Ave.	
**Deficient**	**<80% LPA**	670.250- 792.250	7	1.148	11.480	3.305	0.265	1663.750- 2010.250	9	1.005	11.480	3.291	0.281	255.333- 454.167	13	1.148	11.480	3.704	0.602	265.000- 524.166	9	1.702	11.480	4.462	0.416
**Below** **Normal**	**80–90%** **LPA**	825.333- 887.500	5	1.702	9.238	4.351		2038.750- 2237.250	7	1.233	9.238	3.492		480.833- 524.167	6	1.698	8.523	3.489		532.166- 574.000	9	1.148	8.523	3.193	
**Normal**	**90–110%** **LPA**	926.833- 1089.916	18	1.005	8.523	3.700		2340.500- 2764.500	14	1.181	8.523	4.034		543.500- 627.833	7	2.056	6.587	3.672		598.833- 720.000	7	2.140	4.798	2.961	
**Above** **Normal**	**110-120%** **LPA**	1124.333- 1198.585	6	1.630	5.967	2.889		2780.750- 2848.750	5	1.623	5.967	3.033		660.833- 697.333	6	1.991	4.798	2.970		737.000- 781.833	5	1.560	6.587	2.923	
**Excess**	**>120% LPA**	1253.750- 1441.083	6	1.560	3.982	2.172		3085.000- 3960.750	7	1.560	3.182	2.304		723.000- 960.667	10	1.005	8.485	2.924		792.833- 1029.333	11	1.005	8.485	3.133	

## Discussion

Associations between climatic indicators and vector-borne diseases have been established within many diseases, including VL ( Chen *et al.*, 2012; Dhimal *et al.*, 2015; Malaviya *et al.*, 2011; Perry *et al.*, 2013; Thomson *et al.*, 1999; Thomson *et al.*, 2005; Tiwary *et al.*, 2013). Predictors of *Leishmaniasis spp*. transmission cycles and sensitivity to meteorological and climatic variables is known to vary spatially; dependent on a range of factors including species composition, host competence, contact rates, vector competence, sensitivity to weather and other environmental stressors ( Chaves *et al.*, 2008; Lewnard *et al.*, 2014). These factors however also remain essential for developing early warning systems to prevent epidemics. Due to the lack of spatial and temporal open access granular case data, there are a limited number of studies investigating the interaction of climatic variables and VL in Bihar. Previous studies have predominantly focused on district level climatic trends identifying various monthly or annual temperature, relative humidity and rainfall measurements as the key variables affecting VL transmission ( Malaviya *et al.*, 2011; Napier, 1926; Sivaramakrishnaiah & Ramanatham, 1967; Tiwary *et al.*, 2013). This is the first study to look at historical data spanning 43 years and include climatic variables during specific month periods estimated to impact transmission, namely seasonal sand fly abundance and monsoon period ( Picado *et al.*, 2010).

In this study, four statistically significant negative associations were detected between *Average Temperature* and VL incidence ( *RR= 0.682, p=0.0186* ( *Annual*) and *RR=0.796, p=0.0494* ( *Pre-Sand fly Peak*)) and *Maximum Temperature* and VL incidence ( *RR=0.667, p=0.003 (Annual)* and *RR= 0.814, p=0.0501* (near significant - *Pre-Sand fly Peak*), using GOB data ( Table 2). However after considering the FD correction for multiple testing, only *Maximum Temperature* and VL incidence retained a significant association (0.0360). Overall this shows that risk is reduced with each unit increase of temperature. This could be due to human behavioural patterns, such as more people sleeping outside in warmer temperatures, unfavourable temperatures for optimal sand fly emergence or other trends associated with the vector and transmission dynamics. This is the first time that an association with temperature has been suggested to VL incidence for the *Pre-Sand fly Peak* time period. Annual humidity and VL incidence when fitted to climate anomalies (1977–2013) and adjusted for a decreasing temporal trend in incidence, also showed some association (p=0.0398) however only when corrections for multiple testing were not considered. This suggests that *Annual Humidity* could also play a similar key role in VL transmission to *Annual Maximum Temperature*: influencing human sleeping behaviour.

Historical research indicated that the factors favouring the development of an epidemic would include: altitude below 609.60m above sea level, greater than 1270mm annual rainfall, greater than 70% mean humidity, presence of alluvial soil, maximum temperature below 37.78°C, with diurnal variation less than -6.666°C, abundant vegetation and subsoil water, and a rural environment ( Napier, 1926; Sivaramakrishnaiah & Ramanatham, 1967). Bihar is estimated to be 52.73m above sea level, and maximum temperature was below 37.78°C across all time periods ( Table 2), however rainfall during years of high VL incidence was considerably lower than previously suggested ( Table 4). Other criteria such as “abundant vegetation and subsoil water” cannot be quantified to form a comparable variable within this study. Given the changes in global climate, these historical guidelines on optimal conditions for a VL epidemic may no longer be suitable.

More recent research conducted by Malaviya *et al.* ( Malaviya *et al.*, 2011) showed associations between monthly VL incidence, rainfall and specific humidity, but contrary to this study, found no clear association between incidence and average or maximum temperature. A study conducted in the Gangetic plain, which includes VL endemic West Bengal and Bangladesh, suggested optimal meteorological factors for VL incidence included air temperature of 25.0–27.5°C, relative humidity of 66–75% and annual rainfall between 1000 and 1600 mm ( Bhunia *et al.*, 2010). Due to a lack of available open access data, only annual rainfall data can be compared to findings by Bhunia *et al.* ( Bhunia *et al.*, 2010): rainfall during the periods of high VL incidence fell below the suggested optimal range (1992: 834.667mm, 1991: 670.250mm).

The term epidemic is generally referred to as a sudden increase in the number of cases, beyond what is normally expected within the population of a specific area ( CDC, 2016). The underlying assumption when identifying an epidemic is that the data collection methods adopted are comparable in accuracy and precision. When including GOB&I data, the highest VL incidences were in 1977 and 1991, and historically they have been referred to as “epidemics” ( Bora, 1999; Thakur, 1984b; Thakur, 2007; Thakur *et al.*, 2008). Data forming the basis for these suspected epidemics were collected through active case detection surveys. Such surveys typically detect higher levels of incidence than those recorded through normal passive reporting channels and highlight the need for better case reporting mechanisms. Comparisons between GOB and GOB&I data suggest that case numbers recorded through standard reporting channels in 1977 and 1991 were under-reported (81.41% and 76.15% respectively). Under-reporting of VL in Bihar has been documented since 1977, with most recent reports up to 2006 ( Bora *et al.*, 1994; Singh *et al.*, 2010; Thakur, 1984b). In 2010, it was reported that a “substantial proportion” of patients were visiting private laboratories for diagnosis, which could explain underreporting documented for VL ( Hasker *et al.*, 2010). The quality of passive case reporting may have fluctuated greatly over the years, so a single correction factor cannot be applied across all years of data without further evidence. While the question remains if these are true epidemics, both surveys did result in the implementation of IRS programmes to control the disease.

When considering the current data set, given that the peaks originate from independent surveys, they may simply be artefacts highlighting the true burden of disease. As reported by Bora *et al*., case data recorded by GOB typically includes cases recorded through government health facilities only as private practitioners treating for VL fail to report cases they have treated to the health authorities ( Bora *et al.*, 1994). This was also the issue faced by Dye and Wolpert when modelling the Assam epidemics of 1875 and 1950; where poor quality data limited the findings for the cause behind the second epidemic ( Dye & Wolpert, 1988). Given the discrepancies in data and sources, further evidence is required to confirm the presence of a VL disease cycle.

The anthroponotic nature of the disease in India, suggests records of PKDL could provide additional evidence to explain the peaks seen in 1977 and 1991 ( Addy & Nandy, 1992). Open access case data sources used within this analysis, provided the number of deaths and cases only, restricting further exploration using this variable.

Historically VL vector monitoring within Bihar has been sporadic and limited by lack of spatial and temporal granularity ( Dinesh *et al.*, 2001; Malaviya *et al.*, 2011; Picado *et al.*, 2010; Tiwary *et al.*, 2013). This has prevented the robust multi-annual evaluation of *P. argentipes* abundance peaks, physiology status and transmission effectiveness. For effective retrospective analysis to understand disease incidence, understanding the vector is crucial, furthermore climatic parameters can be further refined based on vector behaviour.

Time periods used within this study were identified as periods of high sand fly activity or adverse weather. The months included within the *Sand Fly Peak* time period (March - June and October - November) have been shown previously to coincide with months of high case numbers ( Dinesh *et al.*, 2001). This relationship needs further exploration, through spatial models, if monthly granular data were available, to further understand disease trends and outbreak prediction.

The available published data describes Bihar’s primary intervention against VL, IRS, as a dichotomous variable, limiting the understanding of its impact on the natural disease patterns. The VL incidence between IRS and non-IRS years is very similar when considering the overall dataset and interestingly increases (3.590) during years of IRS in comparison to years of non-IRS (3.000), suggesting that associations are potentially being masked by the lack of detail about the intervention. Other IRS programme indicators, such as spray quality, would also supplement understanding the success of IRS round, allowing for a robust statistical model to be developed ( Coleman *et al.*, 2015).

Since the start of the VL control efforts strategies adopted for case finding have changed, with the most concerted efforts ongoing considering the current elimination target. Accredited Social Health Activists (ASHAs) today play a key role in promoting healthcare activities such as immunisation, reproductive and child health and detection of VL cases ( Das *et al.*, 2016). Research has shown this incentive based approach for identifying VL patients led to an improved referral rate, translating into early detection of cases and a long-term drop in case numbers ( Das *et al.*, 2016).

The Indian VL elimination programme have taken significant steps to improve case data quality over recent years: increasing VL awareness, providing incentives to local health workers (ASHAs) and implementing district level tracking systems. For the VL elimination programme this means that to sustain the gains made a system such as reactive IRS as seen in other diseases must be established, or, the levels of VL will increase again as observed in Figure 1 ( Coleman *et al.*, 2008). Using the current WHO standards for initiating alerts in India, as in Brazil, after months when the incidence has been double the monthly average, is likely to be a sub-optimal criterion with limited infrastructure and funding to ensure rapid response: particularly after the elimination target has been achieved and the disease is no longer considered a public health concern ( Coleman *et al.*, 2008; Lewnard *et al.*, 2014). A comprehensive understanding of the causative factors for disease trends is required to develop a suitable outbreak detection and response system within the VL Indian context.

## Declarations

### Data availability

Data underlying this study is available from Open Science Framework: http://doi.org/10.17605/OSF.IO/2T9DX ( Deb *et al.*, 2018)

## Supplementary material

Supplementary Table 1: Data sources accessed

Supplementary Table 2: Summaries of the models fitted to climate anomalies for the period 1977–2013.
