# Supplementary material for: Visceral leishmaniasis cyclical trends in Bihar, India – implications for the elimination programme
Source: Gates Open Res. 2018 Feb 21;2:10. [Version 1] doi: 10.12688/gatesopenres.12793.1 (PMC6139379; doi:10.12688/gatesopenres.12793.1)
Supplement: Supplementary file 1 [file gatesopenres-2-13854-s0000.tgz › 17a34f89-699d-440a-bd05-604063a4cbcc.docx]

| **Data** | **Data Origin** | **Source** | **Accessed Date** |
| --- | --- | --- | --- |
| **Case Data** | National Vector Borne Diseases Control Programme, Ministry of Health and Family Welfare | Kala-azar Cases and Deaths in the Country since 2010 (http://nvbdcp.gov.in/ka-cd.html) | 30 June 2016 |
|  |  | Kala-azar Cases and Deaths in the Country since 2007 (http://nvbdcp.gov.in/ka-cd.html) | 08 December 2014 |
|  |  | Sanyal RK, Banerjee DP, Ghosh TK *et al.* A longitudinal review of kala-azar in Bihar. *J Commun Dis* 1979;**11**:149–69. | Published article |
|  |  | Bora D. Epidemiology of visceral leishmaniasis in India. *Natl Med J India* 1999;**12**:62–8. | Published article |
|  |  | Thakur CP, Kumar A, Mitra G *et al.* Impact of amphotericin-B in the treatment of kala-azar on the incidence of PKDL in Bihar, India. *Indian J Med Res* 2008;**128**:38–44. | Published article |
| **Rainfall** | Indian Institute of Tropical Meteorology | <ftp://www.tropmet.res.in/pub/data/rain/iitm-subdivrf.txt> | 08 December 2014 |
|  | Earth System Science Organisation (ESSO)-India Meteorological Department (MoES) | <http://www.imd.gov.in/press_release/20160602_pr_31.pdf> | 08 December 2016 |
| **Temperature and Humidity** | International Research Institute for Climate and Society | <http://iridl.ldeo.columbia.edu/> | 08 June 2016 |
|  | National Centers for Environmental Prediction (NCEP) and the National Centre for Atmospheric Research (NCAR) as part of the NCEP/NCAR Climate Data Assimilation System (CDAS) Reanalysis Project | <http://www.cpc.ncep.noaa.gov/products/wesley/reanalysis.html> | 08 June 2016 |
| **Climate Periodicity** | Published literature | Dinesh DS, Ranjan A, Palit A *et al.* Seasonal and nocturnal landing/biting behaviour for Phlebotomus argentipes (Diptera: Psychodidae). *Ann Trop Med Parasitol* 2001;**95**:197–202. | Published article |

Supplementary Table 1: Data sources accessed
