# Supplementary material for: Visceral leishmaniasis cyclical trends in Bihar, India – implications for the elimination programme
Source: Gates Open Res. 2018 Feb 21;2:10. [Version 1] doi: 10.12688/gatesopenres.12793.1 (PMC6139379; doi:10.12688/gatesopenres.12793.1)
Supplement: Supplementary file 2 [file gatesopenres-2-13854-s0001.tgz › 4e838b56-8b16-491e-817c-ce88eb105b2f.docx]

Supplementary Table 2: Summaries of the models fitted to climate anomalies for the period 1977-2013.

|  |  | Annual | Monsoon Season | Sand Fly Peak | Pre-Sandfly Peak |
| --- | --- | --- | --- | --- | --- |
| Average Temperature | Mean (°C ) | 21.73 | 25.36 | 23.77 | 22.99 |
|  | Range (°C ) | 20.88-23.25 | 24.05-26.77 | 22.85-25.78 | 21.95-24.6 |
|  | s.d. | 0.551 | 0.446 | 0.710 | 0.782 |
|  | Climate Anomaly Coefficient | 0.6783 | 0.9965 | 0.8004 | 0.8014 |
|  | 95% CI | 0.3969-1.1503 | 0.6306-1.5864 | 0.5666-1.157 | 0.5634-1.1437 |
|  | p-value | 0.1493 | 0.9855 | 0.2055 | 0.2456 |
|  | Adjusted p-value | 0.6290 | 0.9855 | 0.6290 | 0.6290 |
|  | Temporal trend | 0.9675 | 0.9669 | 0.9672 | 0.9674 |
|  | 95% CI | 0.9514, 0.9838 | 0.9501, 0.9843 | 0.9510, 0.9836 | 0.9512, 0.9840 |
|  | p-value | <0.0001 | <0.0001 | <0.0001 | <0.0001 |
|  | Adjusted p-value | <0.0001 | <0.0001 | <0.0001 | <0.0001 |
|  | AIC | 800 | 802 | 800 | 800 |
| Maximum Temperature | Mean (°C ) | 28.36 | 28.75 | 31.14 | 30.18 |
|  | Range (°C ) | 27.3-29.75 | 27.21-30.81 | 29.89-32.84 | 28.78-31.97 |
|  | s.d. | 0.645 | 0.644 | 0.795 | 0.866 |
|  | Climate Anomaly Coefficient | 0.7942 | 1.0378 | 0.9685 | 0.8994 |
|  | 95% CI | 0.5254-1.2025 | 0.7666-1.4157 | 0.7348-1.2831 | 0.6816-1.187 |
|  | p-value | 0.2621 | 0.7831 | 0.8243 | 0.4762 |
|  | Adjusted p-value | 0.6290 | 0.9855 | 0.9855 | 0.9524 |
|  | Temporal trend | 0.9670 | 0.9672 | 0.9668 | 0.9671 |
|  | 95% CI | 0.9507, 0.9836 | 0.9503, 0.9846 | 0.9500, 0.9840 | 0.9505, 0.9840 |
|  | p-value | <0.0001 | <0.0001 | <0.0001 | <0.0001 |
|  | Adjusted p-value | <0.0001 | <0.0001 | <0.0001 | <0.0001 |
|  | AIC | 801 | 802 | 802 | 801 |
| Specific Humidity (x1000) | Mean (kg/kg ) | 12.81 | 19.60 | 12.14 | 11.60 |
|  | Range (kg/kg) | 12-13 | 19-20 | 11-13 | 10-12 |
|  | s.d. | 0.394 | 0.495 | 0.639 | 0.541 |
|  | Climate Anomaly Coefficient | 0.5984 | 1.0085 | 0.9242 | 0.9105 |
|  | 95% CI | 0.3542-0.9582 | 0.6802-1.4663 | 0.7073-1.2042 | 0.6082-1.3442 |
|  | p-value | 0.0398* | 0.9652 | 0.5980 | 0.6353 |
|  | Adjusted p-value | 0.4776 | 0.9855 | 0.9530 | 0.9530 |
|  | Temporal trend | 0.9673 | 0.9670 | 0.9673 | 0.9672 |
|  | 95% CI | 0.9515, 0.9835 | 0.9501, 0.9841 | 0.9505, 0.9845 | 0.9503, 0.9843 |
|  | p-value | <0.0001 | <0.0001 | <0.0001 | <0.0001 |
|  | Adjusted p-value | <0.0001 | <0.0001 | <0.0001 | <0.0001 |
|  | AIC | 798 | 802 | 802 | 802 |

* P-value is significant at the 0.05 level
