# Supplementary material for: Visceral leishmaniasis cyclical trends in Bihar, India – implications for the elimination programme
Source: Gates Open Res. 2018 Feb 21;2:10. [Version 1] doi: 10.12688/gatesopenres.12793.1 (PMC6139379; doi:10.12688/gatesopenres.12793.1)
Supplement: Peer review history

# Peer review: Referee response for version 1

**DOI:** 10.21956/gatesopenres.13854.r26550

**Published:** 2018-07-13

**Authors:** Neil Lobo

**Affiliations:** 1 Eck Institute for Global Health, University of Notre Dame, Notre Dame, IN, USA

Potential drivers of the 15 year cyclical trend of Visceral leishmaniasis in India was examined by comparing climactic indicators (rainfall, temperature and humidity) with annual VL case incidence. Relevant monthly periods relating to VL and the vector were defined and used for analysis. The relationship between rainfall and VL incidence, as well as predictors disease were examined. maximum temperature was found to have a negative relationship with VL incidence relating to annual and pre-sandfly peak periods. Relationships between other factors (humidity, rainfall and temperature) and VL incidence were not detected.

 Comments/Questions

- Did the diagnosis (method etc) change over time thereby effecting the incidence?
- Did the GOB reporting change over time thereby effecting the results?
- Could actual Government records of IRS be obtained? I assume the Pubmed/Google references would match the official government records?
- Based on Figure 1, it looks like IRS had a significant effect on VL incidence (reported cases drop off almost immediately and then take about 7 or 8 years to seat going up again. Could the 15 year cycle be based on this?
- I assume active data on incidence is not available except for the 2 years noted?
- Page 5/16: VL peak of 1974 (Bora 1999). This is not in the graph.
- Page 5/16: Gaps in VL incidence data - 1961 to 1976. But the VL peak (see above comment) from 1974 is noted? I might have missed something here.
- Did IRS strategies differ over time? Might this have effected incidence?
- Thank you for mentioning human behavioral patterns.
- The discussion does not mention the 15 year cycle talked about in the abstract.
- Page 10/16: ‘The VL incidence between IRS and non-IRS years is very similar
- when considering the overall dataset and interestingly increases….”. I do not see this trend in the graph (Figure 1). Perhaps just between 1993 and 2014. However, there seems to be an immediate (or almost immediate) drop in incidence immediately following the start of IRS in 1937, 1977-8 and 1992. 
- A follow-on to the above point: might Insecticide resistance effected IRS post 2005?
- Vector data is not present - a limitation/weakness.

I hope this was helpful and constructive.

I have read this submission. I believe that I have an appropriate level of expertise to confirm that it is of an acceptable scientific standard.

# Peer review: Referee response for version 1

**DOI:** 10.21956/gatesopenres.13854.r26318

**Published:** 2018-04-04

**Authors:** Ahmad Ali Hanafi-Bojd

**Affiliations:** 1 Department of Medical Entomology andVector Control, School of Public Health, Tehran University of Medical Sciences (TUMS), Tehran, Iran

This survey is done on a major public health problem in India. Visceral leishmaniasis has a high burden in some parts of this country and is one of neglected tropical disease of diseases with about 500,000 cases annually around the world. Due to the cyclical trend of the disease, the authors tried to understand this pattern by finding the statistical correlation of VL incidence with climatic data. This is a good idea to predict the outbreaks of the disease, given that India is at the elimination phase of VL. To do this study, annual trends of some climate data from 1956-2004 in Bihar State were used to identify potential factors influencing VL incidence. The found statistically significant associations for VL incidence and maximum temperature, and average temperature, when considering annual and pre-sand fly peak time periods. Finally the concluded although the VL program has made significant progress in adopting best practices for improved treatment and vector control; however, open access granular program data for indoor residual spray activities and case detection is required to fully understand the role of climate in disease transmission and potential resurgence.

 Overall this paper is good designed and the results can be useful for implementing in elimination of VL in Bihar. However, in study design I think it could be better if the authors made a correlation between active and passive VL case detection. As they mentioned in the paper, active case detection and therefore the incidence of VL was significantly higher in 1977 and 1991 comparing the passive reports. It is clear that in the active screening, more cases of disease are discovered and treated. This affects both disease statistics and disease cases (that in VL they can act as a reservoir of disease). In my opinion the authors could compare results of active and passive cases detection and then get a coefficient to use in the equation of monthly and annual incidence. In that case, their results about the correlation between VL incidence and climatic factors could be more realistic. Although the effect of vector control program should be mentioned and considered in all calculations.

 Another issue is source data. There seems to be no national database on VL, and authors used published papers and reports. So, true number of cases may be varying. By the way, conduction this survey is appreciated and useful and these deficiencies should be noted and discussed in discussion and conclusion.

I have read this submission. I believe that I have an appropriate level of expertise to confirm that it is of an acceptable scientific standard, however I have significant reservations, as outlined above.

# Peer review: Referee response for version 1

**DOI:** 10.21956/gatesopenres.13854.r26317

**Published:** 2018-03-27

**Authors:** Epco C. Hasker

**Affiliations:** 1 Department of Public Health, Institute of Tropical Medicine (ITM), Antwerp, Belgium

In India VL incidence is considered to be cyclical, with cycles of 15 years. To better understand this pattern, the authors analyzed annual climate data (rainfall, average and maximum temperature and specific humidity) since 1956 and tried to identify associations with reported VL incidence for the state of Bihar. Years were subdivided into monsoon, sand-fly peak and pre-sand-fly peak. Univariate negative binomial models were fitted to asses possible associations between the different climatic factors and VL incidence. To account for multiple testing, the resulting p-values were adjusted using the false discovery rate correction. The only factors significantly associated with VL incidence were maximum and average temperature, for the whole year and for the pre-sand-fly peak period. The authors conclude that to fully understand the role of climate in disease transmission, granular data of IRS and case detection are required.

 Methodologically I think this is all correct, in particular the authors did take into account multiple testing and adjusted their p-values accordingly. Data may be auto correlated though, did the authors take this into account as well?

 The IRS data show that each time there has been a peak, IRS was reintroduced (in 1977, in 1992 and after 2005) and incidence went down again. In the discussion the authors mention that: ‘The VL incidence between IRS and non-IRS years is very similar when considering the overall dataset and interestingly increases during years of IRS (3.590) in comparison to years of non-IRS (3.000)’. On average this may be true as a result of reverse causality but incidence does decrease over the years of each IRS implementation and goes up again when IRS is interrupted. Perhaps it is better to state that: ‘on average VL incidence was higher during IRS years’.

 The issue of under reporting is addressed and the authors do mention that; ‘the quality of reporting may have fluctuated greatly over the years, so a single correction factor across the years of data cannot be applied without further evidence’. For more recent periods there are some estimates available (S.P. Singh et al, 2006, and V.P. Singh et al, 2010). There appears to have been improvement and currently reporting may even be very accurate. It would be worth checking if the associations observed are still significant taking this into account.

 Have the authors considered flooding as a potential factor associated to VL incidence? Emch (2000), exploring this association in Bangladesh states that: ‘People are more likely to contract kala-azar if they live inside, or closer to, a flood-protected area. This may be related to the lack of seasonal inundation of larval habitats in flood-protected areas.’ With or without flood protection, there have been considerable differences in the levels of flooding in Bihar over the years and flooding would be an important additional climatic/environmental factor to include in this study.

 In the conclusion in the abstract the authors state that: ‘open access granular programme data for indoor residual spray activities and case detection is required to fully understand the role of climate in disease transmission and potential resurgence’. From reading the full paper it becomes clear that such data is lacking and is important for properly assessing the association between climatic variables and VL incidence but when reading the abstract the conclusion does not follow logically from the results presented.

 In their final conclusion the authors mention the significant improvements achieved by the Indian VL elimination program in recent years but state that to sustain the gains made, a system such as reactive IRS must be established or the levels of VL will increase again. I do not immediately see how this conclusion is supported by the data presented since (as mentioned earlier) the authors even state that: ‘The VL incidence between IRS and non-IRS years is very similar’.

I have read this submission. I believe that I have an appropriate level of expertise to confirm that it is of an acceptable scientific standard, however I have significant reservations, as outlined above.
